# Metacontrast masking and the cortical representation of surface color: dynamical aspects of edge integration and contrast gain control

**DOI:** 10.2478/v10053-008-0034-z

**Published:** 2008-07-15

**Authors:** Michael E. Rudd

**Affiliations:** Howard Hughes Medical Institute and Department of Physiology and Biophysics University of Washington, Seattle, WA USA

**Keywords:** edge integration, brightness, lightness, achromatic color, brightness induction, masking, metacontrast, paracontrast, type B masking

## Abstract

This paper reviews recent theoretical and experimental work supporting the idea that brightness is computed in a series of neural stages involving edge integration and contrast gain control. It is proposed here that metacontrast and paracontrast masking occur as byproducts of the dynamical properties of these neural mechanisms. The brightness computation model assumes, more specifically, that early visual neurons in the retina, and cortical areas V1 and V2, encode local edge signals whose magnitudes are proportional to the logarithms of the luminance ratios at luminance edges within the retinal image. These local edge signals give rise to secondary neural lightness and darkness spatial induction signals, which are summed at a later stage of cortical processing to produce a neural representation of surface color, or achromatic color, in the case of the chromatically neutral stimuli considered here. Prior to the spatial summation of these edge-based induction signals, the weights assigned to local edge contrast are adjusted by cortical gain mechanisms involving both lateral interactions between neural edge detectors and top-down attentional control. We have previously constructed and computer-simulated a neural model of achromatic color perception based on these principles and have shown that our model gives a good quantitative account of the results of several brightness matching experiments. Adding to this model the realistic dynamical assumptions that 1) the neurons that encode local contrast exhibit transient firing rate enhancement at the onset of an edge, and 2) that the effects of contrast gain control take time to spread between edges, results in a dynamic model of brightness computation that predicts the existence Broca-Sulzer transient brightness enhancement of the target, Type B metacontrast masking, and a form of paracontrast masking in which the target brightness is enhanced when the mask precedes the target in time.

## Introduction

A longstanding tenet of cognitive psychology holds that retinal images are transformed through a series of neural stages from a pixel-based code to higher-order cognitive codes whose properties more closely mirror those of phenomenally perceived objects. The history of the field is, to a large extent, the history of debates concerning the nature of these transformations, the representations corresponding to transformation stages, and their instantiation in neural hardware (Boden, 2006; Lachman, Lachman, & Butterfield, 1979).

Over the last few decades, this serial view of the nature of cognitive information processing in the brain has progressively eroded as neurophysiological data has come to light documenting the importance of parallel processing, lateral connections, and feedback in the construction and maintenance of visual representations. For example, both lateral neural interactions (Gilbert & Wiesel, 1989; Grinvald, Lieke, Frostig, & Hildesheim, 1994; Hirsch & Gilbert, 1991; Kapadia, Westheimer, & Gilbert, 2000; Mizobe, Polat, Pettet, & Kasamatsu, 2001; Stettler, Das, Bennett, & Gilbert, 2002) and re-entrant feedback from higher cortical areas (Lamme, 1995; Lamme, Rodriguez-Rodriguez, & Spekreijse, 1999; Lamme & Spekreijse, 2000; Lamme, Super, & Spekreijse, 1998; Lamme, Zipser, & Spekreijse, 2002; Lee, Mumford, Romero, & Lamme, 1998; Zipser, Lamme, & Schiller, 1996) have been shown to play important roles in the development of neural responses in area V1, an area that was believed a few decades ago to be the home of cells that act as linear spatial filters, passively extracting local edge information.

According to our current understanding, the temporally earliest neural responses in V1 do, in fact, encode the local contrast at edges, but network responses modify these local edge responses later in time (Lamme, 1995; Lamme, Rodriguez-Rodriguez, & Spekreijse, 1999; Lamme & Spekreijse, 2000; Lamme, Super, & Spekreijse, 1998; Lamme, Zipser, & Spekreijse, 2002; Lee, Mumford, Romero, & Lamme, 1998; Zipser, Lamme, & Schiller, 1996). The early, edge-based, responses thus act as “seeds” from which the subsequent network responses self-organize into the complex patterns that form the basis of our conscious perceptions. What is early visual cortex doing with these early edge-based responses? What is the function of the network responses? The results of several recent neurophysiological studies suggest that at least one of the most functional roles played by neural activity in V1 is to support the neural representation of surfaces in the visual environment.

This paper consists of two parts. The first part consists of the description of a model of the cortical computation of surface color based on the idea that color computation involves just a few additional mechanisms beyond the initial edge-based responses in V1, namely, a mechanism that spatially integrates extended edge responses and a mechanism that controls the neural gain applied to these extended edge responses. Both types of mechanisms have been documented to exist in the early cortical visual areas V1 and V2 (Cornelissen, Wade, Vladusich, Dougherty, & Wandell, 2006; Gilbert & Wiesel, 1989; Grinvald, Lieke, Frostig, & Hildesheim, 1994; Haynes, Lotto, & Rees, 2004; Hirsch & Gilbert, 1991; Hung, Ramsden, Chen, & Roe, 2001; Kapadia, Westheimer, & Gilbert, 2000; Kinoshita & Komatsu, 2001; Lee, Mumford, Romero, & Lamme, 1998; MacEvoy, Kim, & Paradiso, 1998; Mizobe, Polat, Pettet, & Kasamatsu, 2001; Rossi & Paradiso, 1999; Rossi, Rittenhouse, & Paradiso, 1996; Sasaki & Watanabe, 2004; Stettler, Das, Bennett, & Gilbert, 2002), although the theory presented here is new.

The second part of the paper consists of a theory of metacontrast masking based on the neural model of surface color computation. There it is shown that the model predicts the existence of Type B metacontrast masking, as well as paracontrast brightness enhancement of the target. While the arguments for the color computation model are well-supported by recent psychophysical and neural data, the metacontrast masking model is more speculative.

### Perceptual evidence for edge integration in achromatic color perception

As a prelude to describing the cortical model of surface color computation, it may be helpful to review some basic facts of spatial color vision. It is well known that the perceived color of a target patch can be strongly influenced by the surrounding spatial context. A chromatic surround tends to induce a tint in the target having a hue complementary to that of the surround (Chevreul, 1839/1967; Goethe, 1810/1970; Hering, 1874/1964; Hurvich, 1981; Jameson & Hurvich, 1964). Similarly, an achromatic gray patch looks darker when it is surrounded by a white surface than it does when it is surrounded by a black surface. These perceptual effects are referred to as *simultaneous color contrast* and *simultaneous lightness (or brightness) contrast*, respectively. In what follows, we will restrict our discussion to achromatic stimuli to keep things simple.

**Figure 1.**
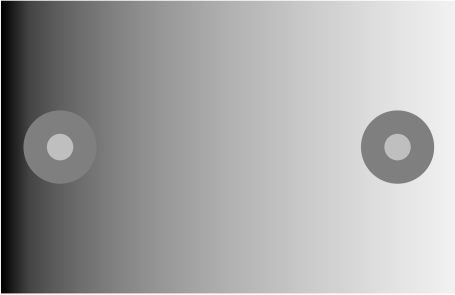
Demonstration of edge integration in lightness perception. The disks and rings on the two sides of the display have identical luminances, but appear lighter when viewed against a dark background. The effect of contrast effect induced by the background affects not only to the ring, which shares a border with the background, but also to the disk, which does not. The disk lightness is also affected by its luminance contrast with respect to the ring (simultaneous contrast). Quantitative studies of lightness matching have shown that the lightness of a target disk is determined by a weighted sum of the local log luminance ratios evaluated at the disk/ring and ring/background borders.

Figure 1 illustrates a perceptual phenomenon that is related to, but not identical with, simultaneous lightness contrast. Here, two identical achromatic disk-and-ring (DAR) stimuli are presented against a background consisting of a luminance gradient. The DAR on the left is positioned against a dark portion of the gradient background and the DAR on the right is positioned against a light portion of the background. The DAR positioned against the dark portion of the background looks lighter than the one that is positioned against the light portion. The key observation for present purposes is that the dark background not only lightens the ring portion of each DAR, which is contiguous with background; it also lightens the central disk, which is not contiguous with the background. We might have imagined otherwise. The background might potentially have affected the appearance of the immediately adjacent surface only; or alternatively the dark background might have lightened the ring on the left which, in turn, might have darkened the left disk. In fact, the latter (false) outcome is predicted by the color model of Jameson and Hurvich (1964) . The fact that the local background affects both the ring lightness and the disk provides an important clue to the nature of lightness processing and is one of the main pieces of support for the lightness model presented below.

The fact that the disks in Figure 1 are affected not only by the immediately adjacent ring but also by the noncontiguous background is consistent with the idea that the lightness of each target disk depends on a *sum* of the luminance contrasts of the disk/ring edge and the ring/background edge corresponding to that disk (Arend, Buehler, & Lockheart, 1971; Gilchrist, 1988; Popa & Rudd, in preparation; Reid & Shapley, 1988; Rudd, 2001; Rudd, 2003a, 2003b; Rudd & Arrington, 2001; Rudd & Zemach, 2004, 2005, 2007; Rudd, in preparation; Rudd & Popa, 2004a, 2004b, 2007; Shapley & Reid, 1985). That is, surrounding the disk with a lighter ring tends to make it look dark because of the contrast of the disk/ring edge, but the contrast of the ring/background edge produces an additional achromatic color induction effect that either lightens or darkens the disk depending on whether the background is lighter or darker than the ring.

I will refer here to the idea that edge contrasts are summed perceptually across space to compute colors as edge integration. The idea of edge integration was introduced into the color perception literature by Edwin Land, whose Retinex theory of color vision (Land, 1977, 1983, 1986, Land & McCann, 1971) was one of the earliest biologically-inspired computational vision models and is still influential in the image processing and human vision literatures.

For the last several years, my colleagues and I have been developing a quantitative edge integration model that makes predictions that are more consistent with perceptual data on lightness matching than are the predictions of Retinex theory. Our model modifies the Retinex edge integration algorithm in some simple but important ways. To understand the model, a little math is required. The reader is reminded of a basic fact of high school mathematics: that multiplying a series of numbers is mathematically equivalent to adding the logarithms of those numbers. It follows that multiplying the local luminance ratios that Retinex computes at luminance borders within the Mondrian across space is mathematically equivalent to summing the logarithms of the edge ratios. The Retinex lightness computation model gives equal weight to each of the log luminance ratios that is summed, but our edge integration model modifies the equal weight rule by postulating the existence of several new principles that determine how the various edges in the scene are weighted in the computing the lightness of a target. The edge weighting rules that we have discovered to date are listed below. These edge weighting rules, when combined with the idea that lightness is computed from a weighted sum of log luminance ratios at edges, form the basis for our edge integration model.

*The edge weighting rules:* 1) Edge weights diminish as a function of distance from the target whose lightness is being computed (Reid & Shapley, 1988; Rudd, 2001; Rudd & Arrington, 2001; Rudd & Zemach, 2004, 2005, 2007; Shapley & Reid, 1985). 2) Edge weights depend on the contrast polarity of the edge whose log luminance ratio is being weighted; that is, the edge weight will be different depending on whether the dark side of the edge, or the light side, points towards the target regions whose lightness is being computed (Popa & Rudd, in preparation; Rudd & Popa, 2004a, 2004b, 2007; Rudd & Zemach, 2004, 2005, 2007). A corollary of this principle is that edges that are perpendicular to the target edges do not contribute to the target lightness at all (Zemach & Rudd, 2007). 3) Edge weights vary as a function of proximities, contrasts, and contrast polarities of other edges in the scene, including, but not restricted to, the target edge (Popa & Rudd, in preparation; Rudd & Popa, 2004a, 2007). 4) Edge weights are subject to top-down attentional control (Rudd, in preparation).

It should be emphasized that these edge-weighting principles are only the ones that have been documented to date. The visual system is quite likely to apply other edge weighting principles, as well. For example, although we have not performed experiments with stimuli in which disparity cues are present to help segment surfaces in depth, we would expect the likelihood of edge integration to depend on whether the target is perceived to be located in the same depth plane as the contextual edges that may potentially contribute to the edge integration computation.

I will next discuss the psychophysical evidence that has led us to propose these edge-weighting principles and I will describe the edge integration model that we have built to instantiate them. Following the exposition of the edge integration model, I will discuss how the model might be extended into the time domain to account for brightness suppression in metacontrast masking.

### Edge weights depend on the distance between the edge and the target

Rudd and Zemach (2004) carried out a study of brightness matching using DAR stimuli consisting of decremental disks surrounded by lighter rings. Before discussing their experiments and results, it is necessary to clarify my use of terminology. Technically, *lightness* refers to perceived reflectance and *brightness* to perceived luminance. But in most studies of “brightness” matching, including that of Rudd and Zemach (2004) , the observer is not given specific instructions to judge either perceived reflectance or perceived luminance, so it is unclear exactly what attribute of the stimulus is matched. The term “brightness” is typically used to refer to the attribute of appearance that is matched in such experiments, although use of the term in such a context does not imply that the observer matched the stimuli in terms of their perceived luminance. As a general rule, the matches made in experiments in which the observers are instructed to match on perceived reflectance are different than those that are made when the observers are instructed to match on perceived luminance or to make a naïve appearance match (Arend & Spehar, 1993a, 1993b; Rudd, in preparation). In my previous work, I have advocated using the term *achromatic color* to refer to stimulus attribute that is matched in naïve matching studies because the term “achromatic color” – like the term “color” – can refer to either perceived surface properties or properties of self-luminous stimuli. Here I will use the colloquial term “brightness” to refer to this attribute in order to avoid the more awkward term “achromatic color” and because brightness is the term that is usually employed to refer to this attribute in the masking literature. In any case, the conclusions that I state in this paper hold regardless of whether the subject is asked to match on perceived reflectance or on perceived luminance, or to perform a naïve appearance match. 

Rudd and Zemach (2004) fitted the results of their naïve appearance matching experiment with an edge integration model based on the assumption that the disk color is computed from a weighted sum of the local log luminance ratios evaluated at the border of the disk and at the outer border of the surround ring. According to this model, the following brightness matching condition should hold at the match point:

(1) $$w_{1}\log\frac{D_{M}}{R_{M}}+w_{2}\log\frac{R_{M}}{B}=w_{1}\log\frac{D_{T}}{R_{T}}+w_{2}\log\frac{R_{T}}{B}$$

where *D_T_* represents the luminance of the target disk, whose brightness was judged in the experiment; *D_M_* represents the luminance of the matching disk, which was adjusted by the observer to achieve a brightness match between the two disks; *R_T_* represents the luminance of the ring surrounding the target, which was varied by the experimenter; *R_M_* represents the constant luminance of the ring surrounding the matching disk; *B* is the constant background luminance, and *w_1_* and *w_2_* are the weights assigned to the log luminance ratios at the inner and outer edges of the surround ring, respectively, by the edge integration algorithm.

Equation (1) has been solved to obtain an expression for the logarithm of the model observer’s matching disk setting as a function of the luminance of the ring surrounding the target (Rudd & Zemach, 2004, 2005). The solution leads to the prediction that a log-log plot of the matching disk luminances versus the luminance of the target ring will be a straight line having a slope equal to *w_2_*/*w_1_* –1. By fitting a linear regression model to a plot of experimental data and estimating the slope, we can estimate the ratio *w_2_*/*w_1_* of the weights associated with the outer and inner edges of the surround ring.

The brightness matching study performed by Rudd and Zemach (2004) using decremental disks yielded weight ratio estimates ranging from 0.21 to 0.36 for four observers (Rudd & Zemach, 2004, Experiment 1). We repeated this experiment with rings of various widths and found that the weight ratio estimates decreased monotonically with increases in the ring width (Rudd & Zemach, 2004, Experiment 2). This latter result is consistent with the assumption that the weights assigned to edges in the computation of the target color diminish with distance from the test or matching disk. The brightness matching equation (1), which is based on a weighted sum of log luminance ratios, was found to provide a better fit to the data from these experiments than did appearance models based on luminance matching (*w_2_*=*w_1_*), ratio matching (*w_2_* = 0; Wallach, 1948, 1963, 1976), or a weighted sum of the local Michelson contrasts evaluated at the inner and outer edges of the ring (Reid & Shapley, 1988; Shapley & Reid, 1985).

### Edge weights depend on the contrast polarities of the perceptually integrated edges

In a follow-up study (Rudd & Zemach, 2005), observers performed brightness matches with disks that were luminance increments with respect to their surround rings. The disks and rings were identical in size to those used in Experiment 1 of our experiment with decremental disks. The weight ratio estimates obtained in the study with incremental targets ranged from .64 to .95. These weight ratio estimates can be converted to quantitative measures of the magnitude of the brightness induction from the surround: that is, to measures of the degree to which manipulating the surround luminance influences the target brightness (Rudd & Zemach, 2005). According to this measure, incremental targets were subject to a 5-36% brightness induction effect from the surround, where a 100% contrast effect is defined as a match based on equal disk/ring luminance ratios and a 0% contrast effect is defined as a match based on the disk luminance alone. By comparison, the matches performed with decrements indicated a 60-80% surround induction effect. The magnitude of the contrast effect obtained when the targets were increments was both considerably smaller (3.25 times smaller, on average) and more variable than that of the contrast effect obtained when the targets are increments.

From the point of view of edge integration theory, the main difference between the stimuli used in the two studies was the contrast polarity of the disk edge. In the 2004 study using decremental targets, the disk edge was dark-inside, while in the 2005 study using incremental targets, the disk edge polarity was light-inside. In both studies, the outer edge of the surround ring was light-inside. The differences between the weight ratio estimates obtained in the two studies might therefore be attributed to differences in the relative weights given to dark-inside and light-inside edges. Taking this as our working hypothesis, we conclude that the weights associated with edges of the light-inside type are, on average, about 3.25 times smaller than the weights associated with edges of the dark-inside type, all other things being equal. This difference quantifies the well-known asymmetry in the magnitudes of the surround induction effects obtained in previous studies of achromatic color matching performed with incremental and decremental stimuli (Agostini & Bruno, 1996; Bressan & Actis-Grosso, 2001; Gilchrist, Kossyfidis, Bonato, Agostini, Cataliotti, Spehar et al., 1999; Heinemann, 1955, 1972; Hess & Pretori, 1884/1970; Jacobsen & Gilchrist, 1988; Kozaki, 1963, 1965; Wallach, 1948, 1963, 1976; Whittle & Challands, 1969).

### The role of contrast gain control in achromatic color perception

In addition to the evidence for edge *integration* in brightness perception cited above, we have also found evidence for interactions between edges, where the term edge *integration* refers to the presence of an additional term in the brightness matching equation — not included in Equation (1) — involving products of log luminance ratios evaluated at separate edges; for example, a term of the form *k* log(*D*/*R*)log(*R*/*B*), where *k* is a constant. Multiplicative terms of this sort must be added to the brightness matching equation to account for statistically-significant quadratic components seen in the log-log plots of matching disk luminance versus test ring luminance obtained in matching experiments performed with both incremental and decremental DAR stimuli (Rudd & Zemach, 2004, 2005, 2007; Vladusich, Lucassen, & Cornelissen, 2006). That is, log *D_M_* vs log *R_T_* plots are better fit by regression models based on parabolic curves than by models based on straight lines, although the parabolic curves are sometimes sufficiently straight to be well-approximated as straight lines. In some studies and for some observers, the amount of variance explained by the quadratic component was as small as a fraction of a percent, while in other studies and for other observers, the amount of variance explained by the quadratic component was large.

The curvature in the log *D_M_* vs log *R_T_* tends to be most pronounced when the DAR stimuli are presented against a light background and thus when the contrast polarity outer edge of the surround ring is dark-inside (Rudd & Zemach, 2007). Under these conditions, the curvature of the log *D_M_* vs log *R_T_* plot may be large enough to rule out the use of a linear approximation. When the background field is dark, deviations from the linear model are typically negligible, although such deviations can be detected using statistical methods (Rudd & Zemach, 2004, 2005, 2007; Vladusich, Lucassen, & Cornelissen, 2006).

The need to include edge interaction effects in the brightness matching equation was first noted by Rudd (2001; Rudd & Arrington, 2001), who proposed a mechanistic model to account for the edge interactions. Like the model corresponding to Equation (1), which does not include such interactions, the model of Rudd and Arrington assumes that the brightness of a target region is computed from a weighted sum of brightness induction signals derived from multiple borders. However, the Rudd-Arrington model makes the further assumption that spatially spreading color filling-in signals originating from remote edges are partially “blocked” by the target border.

The hypothesis was originally proposed to account for data from matching experiments carried out with target disks surrounded by two rings, rather than one ring. But the idea behind the model is perhaps best illustrated using the example of a test disk surrounded by a single ring (Rudd & Zemach, 2007). According to the edge integration model discussed above in the context of the experiments of Rudd and Zemach – the model without blockage – the brightness of such a disk is computed from a weighted sum of the log luminance ratios associated with the inner and outer borders of the ring. That is

(2) $$\Phi =w_{1}\log\frac{D}{R}+w_{2}\log\frac{R}{B}$$

where the symbol Φ denotes the magnitude of a neural signal on which judgments of the disk appearance are based. The blockage model modifies Equation (2) so that the *effective* weight associated with the outer border of the surround ring depends on the log luminance ratio of the disk/ring border. The modified equation for the magnitude of the neural signal associated with the disk brightness is

(2a) $$\Phi =w_{1}\log\frac{D}{R}+w_{2}\left(1-\beta \left|\log\frac{D}{R}\right|\right)\log\frac{R}{B}$$

Rudd and Arrington suggested that Equation (2a) is the signature of an underlying brightness filling-in mechanism in which the brightness induction signal originating from the outer ring edge is partially blocked, in a contrast-dependent manner, by the inner ring edge. According to this interpretation of equation (2a), the magnitude of the brightness induction signal that is produced by the outer border of the ring and contributes to the disk brightness would, in the absence of blockage, be *w_2_*log(*R*/*B*) if it were not for the fact that a percentage β|log(*D*/*R*)| of this induction signal is prevented from reaching the disk by a contrast-dependent blockage at the disk/ring border.

Rudd and Arrington proposed the blockage interpretation in the context of a filling-in theory of brightness induction. Brightness filling-in theories assert that induction signals originating from borders diffuse like dye within a spatiotopic cortical map of the retinal image to fill in regions lying between borders (Arrington, 1994; Cohen & Grossberg, 1984; Gerrits, de Haan, & Vendrik, 1966; Gerrits & Timmermann,1969; Gerrits & Vendrik,1970; Grossberg & Mingolla, 1985; Grossberg & Todorovic, 1988; Paradiso & Hahn, 1996; Paradiso & Nakayama, 1991; Pessoa, Thompson, & Noe, 1998; Rossi & Paradiso, 1996, 1999; Sasaki & Watanabe, 2004). According to such theories, edge-based induction signals are blocked – or, in the case of the Rudd-Arrington model, they are partially blocked – by other borders that these spreading neural signals encounter while diffusing within a cortical map of the visual scene.

The absolute value sign appearing in the term for the percent of the signal that is blocked in Equation (2a) is necessitated by the fact that the proportion of the filling-in signal that is blocked is assumed to be physiologically instantiated as a firing rate of a cortical neuron and firing rates must necessarily be positive. The firing rate is, in turn, assumed to be proportional to the log luminance ratio of the disk-ring edge that is encoded by the edge detector neuron whose neural activity blocks the filling-in signal. The log luminance ratio can be either positive or negative depending on the contrast polarity of that edge, but the firing rate must be positive; so the absolute value sign is required to map the log luminance ratio of the blocking edge into the firing rate associated with a neuron that encodes the edge contrast. The edge contrast polarity is assumed to be implicitly encoded by the polarity preference of the edge detector neuron that does the blocking (labeled line). The proportionality constant β determines the percentage of the filling-in signal that is blocked as a function of the log luminance ratio of the disk-ring edge. This constant is referred to as the *blocking coefficient* .

Rudd and Zemach (2007) fit Equation ((2a) to the data from brightness matching experiments carried out with DAR stimuli having all four possible combinations of inner and outer ring edge contrast polarities. In addition to the matching data from the experiments cited above, in which DAR stimuli with incremental and decremental disks were presented against dark backgrounds, Rudd and Zemach analyzed data from two new matching experiments in which incremental and decremental DARs were presented against light backgrounds. 

Although brightness matching equation (2a) was found to provide an excellent fit to the data from all four experiments, the sign of the blocking coefficient was found to vary with the contrast polarity of the inner ring border. The fact that the “blocking” coefficent is sometimes negative rules out a mechanistic interpretation of the equation in terms of the partial blocking of a diffusing color signal, because such an interpretation would then require that a negative proportion of the induction signal originating from the outer edge be blocked in those conditions where β is negative, which is clearly nonsensical.

Because of this problem, my colleagues and I (Popa & Rudd, in preparation; Rudd & Popa, 2004a, 2004b, 2007, Rudd & Zemach, 2007) have recently proposed an alternative neural mechanism to account for the edge interaction effects that have now been seen several studies (Rudd & Arrington, 2001; Rudd & Zemach, 2004, 2005, 2007; Vladusich, Lucassen, & Cornelissen, 2006). This alternative mechanism explains the edge interaction effects on the basis of a cortical gain control process by which the spike rates of cortical edge detector neurons in the cortical map of the image are modified by the activities of other nearby edge detector units. The theory combines this cortical gain control mechanism with a neural edge integration process that is assumed to occur at a later stage of visual processing. This model is able to account for the results of all of the brightness matching studies that have analyzed to date. In what follows, I will refer to this model that combines edge integration and contrast gain control as the *contrast gain control model*, for short.

**Figure 2.**
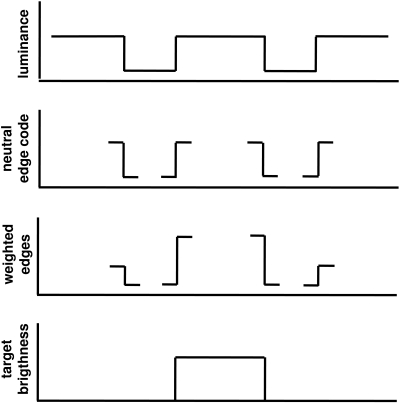
Schematic diagram illustrating the stages involved in computing the brightness of a light target surrounded by a dark ring viewed against a light background, according to the edge integration model with contrast gain control. The graph at the top of the figure, labeled “luminance” shows a one-dimensional cross-section of the stimulus profile. This stimulus comprises the input to the edge integration computation. The graph below that, labeled “neural edge code,” shows the locations in which edge detector neurons encode the presence and the log luminance ratios of luminance borders in the input image. Separate neurons are assumed to encode edges having different contrast polarities. The third graph in the figure illustrates the fact that the responses of the edge encoding units that are nearer to the target disk are weighted more heavily in the computation of target brightness than are the response of remote edge encoding units. Contrast gain control acting between the inner and outer edges of the surround ring also contributes to the steady state values of the weights applied to the two edges. The bottom graph shows the profile of the target brightness, which is computed from the weighted sum of the disk/ring and ring/background edges. The inner edge, which has a light-inside contrast polarity, lightens the target to a degree that depends on the weighted log luminance ratio of the inner edge. The outer edge, which has a dark-inside contrast polarity, darkens the target to a degree that depends on the weighted log luminance ratio of the outer edge. Since the absolute magnitude of the weighted log luminance ratio at the inner edge is larger than the absolute magnitude of the weighted log luminance ratio at the outer edge, the target will appear light, rather than dark, relative to the background.

The contrast gain control model differs from the blockage model by assuming not only that the effect of an induction signal originating from the outer ring edge can be influenced by the local contrast of the disk edge (as in the blockage model), but that an induction signal derived from the disk edge can also be influenced by the local contrast of the outer ring edge (Rudd & Zemach, 2007). The contrast gain control model further assumes that the gain control is strongest when the edges are close together and diminishes in magnitude as a linear function of distance (Rudd & Popa, 2007). A diagram illustrating the various stages of neural processing contributing to the computation of brightness in the contrast gain control model is presented in Figure 2.

These assumptions have been formalized mathematically (Popa & Rudd, in preparation; Rudd & Popa, 2007) and are expressed in the following equation, which asserts that the brightness of a disk surrounded by a ring of homogeneous luminance is determined by the expression:

(3) $$\Phi =w_{1}^{*}\left(1+v_{2\to 1}\left|\log\frac{R}{B}\right|{\left[1-\frac{d}{s_{2\to 1}}\right]}^{+}\right)\log\frac{D}{R}+w_{2}^{*}\left(1+v_{1\to 2}\left|\log\frac{D}{R}\right|{\left[1-\frac{d}{s_{1\to 2}}\right]}^{+}\right)\log\frac{R}{B}$$

where *w*_1_^*^ and *w*_2_^*^are the weights that would be assigned to the inner and outer ring edges in the achromatic color computation in the absence of any gain-modulating influence (i.e., if there were no other nearby edges, or the log luminance ratios of the nearby edges were equal to zero); *d* is the ring width (i.e., the distance between the inner and outer ring edges); the symbol [ ]+ signifies the half-wave rectification operation, which returns either the value of the expression in brackets or the value zero, whichever is larger; and the model parameters *v_2→1_*, *v_1→2_*, *s_2→1_*, and *s_1→2_* are interpreted as follows. The parameter *v_i→j_* specifies the rate at which the magnitude of the gain applied to edge *j* by a gain control signal originating from edge *i* grows as a function of the absolute value of the local log luminance ratio of edge *i*. The sign of *v_i→j_* determines whether the gain-modulating signal directed from edge *i* to edge *j* acts to either increase (plus sign) or decrease (negative sign) the gain applied to neurons encoding the log luminance ratio of edge *j*. The parameter *s_i→j_* represents the maximum spatial spread of the neural gain-modulating signal directed from edge *i* to edge *j*. The expression within the half-wave rectification brackets models the fact that the magnitude of the contrast gain modulation decreases as a linear function of the distance between the edges. The half-wave rectification ensures that no gain modulation will occur when this distance exceeds the spatial range *s_i→j_* of the gain control directed from *i* to *j*.

As in the blockage model, the log luminance ratios log(*D*/*R*) and log(*R*/*B*) are assumed in the contrast gain control model to be neurally instantiated as firing rates. Again, these firing rates must necessarily be positive and are often modeled by half-wave rectifying the output of a model spatial receptive field. It follows that different cortical neurons will encode the log luminance ratio at an edge depending on the contrast polarity of that edge. For example, negative log luminance ratios will be encoded by neurons responding to dark-inside edges. When log(*D*/*R*) is negative, we therefore assume that the log luminance ratio of the disk-ring edge is encoded by a half-wave rectifying neuron whose firing rate represents the quantity [log(R/*D*)]+, which in this case is the same thing as the absolute value of log(*D*/*R*), and that the firing rate is given a negative synaptic weight in the neural edge integration computation. In this way, the quantity log(*D*/*R*) can be represented by synaptically-weighted neural firing rates, even though the rates are positive and the log luminance ratio is negative. That is, the positive firing rate of an edge-detector neuron will inhibit the activities of the higher-order neurons that encode the disk brightness or, equivalently, excite higher-order neurons that encode the disk *darkness*.

When log(*D*/*R*) is positive, on the other hand, the log luminance ratio of the disk-ring edge will be encoded by a different neuron: a neuron whose receptive field is in the same location as that of the first neuron but whose firing rate represents the quantity [log(*R*/*D*)]^+^. The response of this second neuron will be given a positive weight in the edge integration computation.

Equation (3) also involves terms with absolute values of log luminance ratios, such as |log(*D*/*R*)|. These terms also must be neurally instantiated in order to realize the contrast gain control mechanism proposed in the model. Again, the firing rates of two different edge detector units, having receptive fields located at the same retinal position and being sensitive to edges having the same orientation, will represent either the mathematical quantity [log(*D*/*R*)]^+^ or the mathematical quantity [log(*R*/*D*)]^+^, depending on whether the edge detector responds preferentially to edges of the light-inside edge or the dark-inside type.

Because these cortical neurons half-wave rectify their inputs, whenever one of these two neurons fires the other will be silent. It follows that the outputs of the pair of neurons must be summed in order to compute the quantity |log(*D*/*R*)|, independent of the contrast polarity of the disk-ring edge, as required by Equation (3). Similar considerations apply to the computation of the log luminance ratios and the absolute values of the log luminance ratios corresponding to all the edges in the image. Thus, we see that neural mechanisms described above would suffice to instantiate the mathematical operations required by Equation (3) in a neurally-plausible manner.

Popa and Rudd (in preparation) have developed a computer program for the purpose of simulating this neural model of edge integration and contrast gain control. We have used our program to simulate the data from some brightness matching experiments in which the luminance of a test square surrounded by a frame and the frame width were independently varied. By fitting the model to this new data, we have discovered that the sign of the gain modulation term (that is, whether the contrast gain control originating from any particular edge detector unit acts to increase or to decrease the gain of a neighboring unit) depends on the preferred contrast polarities of the two units and, therefore, on the contrast polarities of the luminance borders that drive them.

### Neurophysiological evidence for edge-based color induction mechanisms in areas V1 and V2

It has been known since the early single-cell recording studies of Hubel and Wiesel (1959, 1968, 1977) that neurons in area V1 respond preferentially to properly oriented bars or edges presented with the classical receptive fields of these neurons. The results of recent physiological studies indicate that neurons in V1 (and V2) play a much larger role in perceptual organization and surface perception than the picture of neurons in these areas as mere edge detectors suggests. For example, it has been shown that neurons in these cortical areas are sensitive to Gestalt stimulus properties such as figure-ground segmentation (Lamme, 1995; Lamme, Rodriguez-Rodriguez, & Spekreijse, 1999; Lee, Mumford, Romero, & Lamme, 1998; Zipser, Lamme, & Schiller, 1996) and border ownership (Friedman, Zhou, & von der Heydt, 2003; Qiu & von der Heydt, 2005; von der Heydt, Friedman, & Zhou, 2003; von der Heydt, Zhou, & Friedman, 2003; Zhou, Friedman, & von der Heydt, 2000).

The role played by V1 and V2 in the representation of surface lightness, brightness, and color is less clear, but we know that at least some of the neurons in these areas respond to modulation of border contrast outside of their classical receptive fields (Cornelissen, Wade, Vladusich, Dougherty, & Wandell, 2006; Kinoshita & Komatsu, 2001; MacEvoy, Kim, & Paradiso, 1998; Rossi & Paradiso, 1999; Rossi, Rittenhouse, & Paradiso, 1996; Vladusich, Lucassen, & Cornelissen, 2006), which suggests that these neurons help to mediate spatial color induction from borders and may even form the stage of neural processing that is most closely associated with the perceptual filling-in of surface color (Haynes, Lotto, & Rees, 2004; Hung, Ramsden, Chen, & Roe, 2001; Kinoshita & Komatsu, 2001; Lee, Mumford, Romero, & Lamme, 1998; MacEvoy, Kim, & Paradiso, 1998; Rossi & Paradiso, 1999; Rossi, Rittenhouse, & Paradiso, 1996; Sasaki & Watanabe, 2004).

Of special interest from the standpoint of edge integration theory is a recent fMRI study by Cornelissen et al. (2006) showing long-range edge responses that span a distance of about 18 mm on the cortical surface, which is well beyond the spatial limits of the classical V1 and V2 receptive fields. In terms of visual angle, the span of these long-range edge response is about 5-10 deg, which corresponds roughly to the spatial spread of the achromatic color induction effects measured in psychophysical studies (Cole & Diamond, 1971; Diamond, 1953, 1955; Dunn & Leibowitz, 1961; Hong & Shevell, 2004; Leibowitz, Mote, & Thurlow, 1953; Reid & Shapley, 1988; Rudd & Zemach, 2004).

Cornelissen et al. suggested that long-range edge responses in V1 and V2 might subserve the function of either edge integration, or “contextual influences on the edge,” or both. This raises the possibility that at least some of the neural processes predicted by our contrast gain control model may be carried out in areas V1 and V2. The extended edge responses might represent the activities of neural processes that “reach out” to adjust the weights of other nearby edge detector units (contrast gain control), or they might correspond to the edge-based color induction signal itself, or they might reflect a mixture of these two types of activity.

The spatial summation of edge-based induction signals that is required to account for the achromatic color matching results has not been explicitly investigated by neurophysiologists. This summation might also take place in either V1 or V2. Or it might be carried out at a higher level of the visual system. Area V4 seems a likely site of the neural edge integration operation, since the outputs of V1 and V2 neurons project to V4 and the large receptive fields of V4 neurons would allow for a spatial summation over many degrees of visual angle, as is required to account for the psychophysical data. The latter suggestion is also consistent with the proposition, put forth by several previous investigators, that V4 plays a special role in color constancy (Bartels & Zeki, 2000; Clarke, Walsh, Schoppig, Assal, & Cowey, 1998; Kennard, Lawden, Morland, & Ruddock, 1995; Kentridge, Heywood, & Cowey, 2004; Smithson, 2005; Walsh, 1999; Zeki, Aglioti, McKeefy, & Berlucchi, 1999; Zeki & Marini, 1998), since the purpose of edge integration is to help achieve constancy (Land, 1977, 1983, 1986; Land & McCann, 1971).

In the remainder of this paper, I will extend this model into the time domain to devise a dynamic brightness perception model that accounts for the existence of both metacontrast and paracontrast masking phenomena.

### Possible relationship of metacontrast to edge integration and contrast gain control

In this section of the paper, I will discuss how the brightness computation model presented above might relate to metacontrast masking. I will not present any new masking data, but I will propose a theory of metacontrast and discuss how this theory could be tested in future experiments.

In metacontrast masking, a mask that follows the target in time suppresses the target brightness. The mask often (but not always) has its greatest effect when it follows the target by a delay of about 50-100 milliseconds. When the target brightness is measured as a function of the temporal delay between the target and the masking stimulus, a U-shaped brightness function is obtained. The U-shaped brightness function is often taken to be one of the characteristic features of metacontrast masking (Alpern, 1953; Breitmeyer, 1984; Breitmeyer & Öğmen, 2006). Although situations do occur in which the brightness function associated with metacontrast masking is a monotonic rather than a U-shaped function of time, I will here restrict my discussion to the special case of U-shaped (Type B) metacontrast masking (Breitmeyer, 1984), leaving it for future work to extend the model presented here to account for monotonic metacontrast masking functions.

Early metacontrast studies typically employed either an oriented bar as the target and flanking bars as the mask (e.g., Alpern, 1953), or a disk as the target and a surround ring as the mask (e.g., Werner, 1935). The potency of the mask was found to be greatest when: 1) the mask followed the target with the correct stimulus onset asynchrony (SOA); 2) the target and mask edges were in close spatial proximity (Alpern, 1953; Breitmeyer, 1984; Fry, 1934; Kolers, 1962; Kolers & Rosner, 1960; Levine, Didner, & Tobenkin, 1967; Stigler, 1926; Weisstein & Growney, 1969); and 3) the mask had a large contrast energy relative to that of the target (Breitmeyer, 1978a; Breitmeyer, 1984; Fehrer & Smith, 1962; Kolers, 1962; Spencer & Shuntich, 1970; Stewart & Purcell, 1974).

Several investigators have noted the special importance of border contour in metacontrast masking (Breitmeyer, 1984; Kolers, 1962; Weisstein, 1971; Werner, 1935). For example, Werner (1935) found that metacontrast was strongest when the borders of the target and mask were most similar. Weisstein (1971) obtained a U-shaped masking curve by masking a small disk target with a larger disk mask. She interpreted her results in terms of the hypothesis that metacontrast entails interactions between edges, rather than interactions between surfaces or objects. 

In fact, many of the same stimulus factors that control the strength of edge interactions in metacontrast displays – e.g., spatial proximity, contour similarity, and border contrast polarity (Becker & Anstis, 2004; Breitmeyer, 1978b) – have also been shown to influence the strength of edge integrations in the perception of static brightness displays (Bindman & Chubb, 2004a, 2004b; Hong & Shevell, 2004; Popa & Rudd, in preparation; Reid & Shapley, 1988; Rudd, 2001, 2003a; Rudd & Arrington, 2001; Rudd & Popa, 2004a, 2004b, 2007; Rudd & Zemach, 2004, 2005, 2007; Vladusich, Lucassen, & Cornelissen, 2006; Zemach & Rudd, 2007). The similarities between the brightness suppression that occurs in metacontrast masking and the contrast gain control phenomena observed in studies using static DAR displays are provocative. These similarities suggest that perhaps both phenomena might be accounted for by the same underlying mechanism or mechanisms.

The theory of metacontrast masking presented here is based on the idea that metacontrast occurs at a stage of neural processing at which edges interact and at which multiple edges may influence the target brightness via the mechanism of edge integration, but at which an object representation has not yet been formed. The fact that metacontrast can occur when the target and mask are presented to separate eyes implies a cortical locus for the interaction (Breitmeyer, 1984; Kolers & Rosner, 1960; May, Grannis, & Porter, 1980; Schiller & Smith, 1968; Stigler, 1926; Weisstein, 1971; Werner, 1940).

Many theories have been advanced to account for the U-shaped metacontrast masking function. Francis (2000) has presented a useful classification of some of these theories (see also Francis & Cho, 2006; Francis & Herzog, 2004). Perhaps the most common type of theory invokes a mechanism whereby an afferent neural signal originating from the mask overtakes in time and inhibits a corresponding neural signal from the target (Breitmeyer, 1984; Breitmeyer & Ganz, 1976; Breitmeyer & Öğmen, 2006; Stigler, 1926). But the “overtake and inhibit” hypothesis is far from universally accepted. Francis considers several other mechanistic accounts of metacontrast, and Reeves (1982) has argued that the U-shaped masking function does not result from a single process — such as inhibition of the target by the mask at some preferred delay — but rather from two separate processes that each produce a monotonic change in the target brightness as a function of the temporal delay between target and mask. 

For an in-depth review of both the basic data on metacontrast and a larger body of theories that have been proposed to account for it, the interested reader is referred to review articles by Alpern (1952) , Weisstein (1972) , Lefton (1973) , Breitmeyer (1984) , Francis (2000) , and Breitmeyer and Öğmen (2006) . 

In the remainder of the present paper, I will confine my remarks to the discussion of the hypothesis that metacontrast phenomena should be viewed as a byproduct of the dynamical properties of brightness computation by human visual cortex and that metacontrast masking results, more specifically, from the dynamics of edge integration and contrast gain control.

### Metacontrast masking from edge integration dynamics

To investigate the possible connection between edge integration and metacontrast, we first need to address the problem of how the edge integration model might be extended into the time domain. For concreteness, we will analyze the metacontrast paradigm introduced by Weisstein (1971), in which a target disk is followed in time by a larger masking disk. This is a particularly simple stimulus display from the standpoint of the edge integration model, since it involves only one target edge and one mask edge. The Weisstein display differs from the static DAR stimuli used in our previous matching experiments only in that a temporal delay is imposed between the onset of the target edge and the onset of the more distant edge. To study metacontrast with such a stimulus, it is best to present the target and masking disks to different eyes; otherwise brightness masking (Turvey, 1973) occurs at brief stimulus onset asynchronies in addition to the Type B metacontrast effect, which is seen at longer SOAs. Thus, a W-shaped masking function is obtained with the target and mask are presented to the same eye (Weisstein, 1971). 

**Figure 3.**
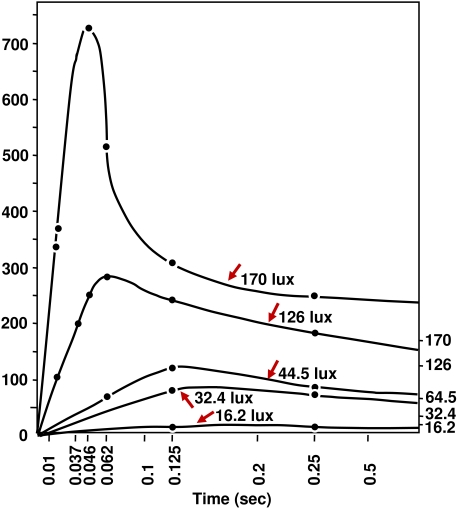
Broca-Sulzer brightness enhancement occurs at stimulus onset for high intensity incremental targets. Here flash brightness is plotted as a function of duration for flashes of different luminances. Data from Hart (1987).

In considering the dynamics of edge integration, it is important to take into consideration the so-called *Broca-Sulzer effect*: the brightness of a flashed stimulus is temporally enhanced at stimulus onset (Alpern, 1963; Boynton, 1961; Breitmeyer, 1984; Broca & Sulzer, 1902, 1904; Stainton, 1928) (see Figure 3). The Broca-Sulzer effect is likely due to transient components of the firing rates of early visual neurons (see, for example, Saito & Fukada, 1986), and is closely related to Crawford masking (Breitmeyer, 1984; Crawford, 1947).

By adding the assumption that the neural responses to edge contrast exhibit such transient components to the other postulates of the edge integration model, we arrive at a model that can account for some of the known properties of metacontrast masking and that also makes testable theoretical predictions. Our main focus will be on applying this model to Weisstein’s masking paradigm in which the target and mask are both disks, so that the target and mask each have a single edge. The response of the model to such a stimulus is much simpler to analyze than is the model response to the more typical masking stimulus in which the target is a disk, the mask is a ring, and there is potential a gap between them. The latter stimulus includes three edges that could produce fairly complex cortical interactions between edge detector units, given that any pairwise combination of edge detector responses may be subject to two-way gain control interactions. Whereas in the case of the Weisstein stimulus we only need to keep track of two gain control signals (the outward and inward directed signals acting between the target and mask edges), a total of six gain control signals could come into play when the masking stimulus is a ring.

According to the edge integration model, when a light target disk (i.e., a target that is a luminance increment with respect to its immediate surround) is presented in isolation against a dark background field, the disk brightness will be completely determined by the log luminance ratio at its border. As a result of the Broca-Sulzer effect, the disk will appear transiently brighter immediately after its onset than it does in the steady state. It is well-known that the steady-state brightness of a disk viewed in isolation obeys Stevens’ brightness law, which states that the brightness of a static target viewed in the dark is proportional to the target luminance raised to approximately the 1/3 power (Rudd & Popa, 2007; Stevens, 1953, 1961, 1967, 1975; Stevens & Marks, 1999). The exponent of the brightness law decreases from about 1/2 to about 1/3 as the flash duration is increased from 0.5 to 1000 msec (Aiba & Stevens, 1964; Raab, 1962; Stevens, 1966; Stevens & Hall, 1966). The exponent of Stevens’ law can be viewed as an “exponential gain” applied to the target (Rudd & Popa, 2007; Whittle, 1994). According to the edge integration model, the gain applied to the target reflects the gain of neural edge detector units in early visual cortex (Popa & Rudd, in preparation; Rudd & Popa, 2004a, 2004b, 2007). These findings all follow from the edge integration model if is assumed that the outputs of the edge detector units that encode the edges of the target exhibit a transient increase in their firing rates at stimulus onset, an assumption that is well-supported by physiology.

**Figure 4.**
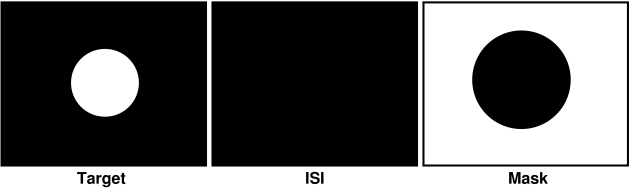
A metacontrast masking paradigm modeled after the experiment of Weisstein (1971). A target consisting of an incremental disk displayed against a dark background is shown to one eye. Following a dark interstimulus interval of variable duration, a masking stimulus consisting of a decremental disk, larger in size than the target disk, is displayed to the contralateral eye. This stimulus differs from Weisstein's in that here the masking disk is dark, whereas in Weisstein's paradigm the target and mask both consisted of bright disks displayed against dark backgrounds. In both experimental paradigms, the target and mask each have only one edge.

Now suppose that a second, larger, masking disk is presented to the eye contralateral to the one that sees the target. Further suppose that the mask is presented after a variable interstimulus interval (ISI) following the target disk presentation, as in Weisstein’s experiment. In her experiment, the target and masking disks were both luminance increments with respect to their immediate surrounds, but we will begin here by analyzing the situation in which the masking disk is a luminance decrement with respect to its surround (i.e., the background field). In this case, the dark side of the mask edge fsaces the incremental disk target (see Figure 4).

When the stimulus onset asynchrony is very short (i.e., SOA = 0), the target and mask onsets are simultaneous. In that case, according to the edge integration model, the target brightness will be determined by a weighted sum of contributions from the target and mask edges, as in the case of a static DAR stimulus. At the onset of both target and mask, the amplitudes of both of these components will be temporarily boosted by the transient neural activity in the edge detector neurons that encode the edges. As these transient activities decay, there may be a change in the target brightness, but this decay will be monotonic in time since the weighted sum of two monotonically decaying functions will also exhibit monotonic decay. The decay of transient activity cannot by itself account for Type B metacontrast masking, since any explanation of Type B metacontrast requires a mechanism that can produce a U-shaped masking function under the appropriate stimulus conditions.

What if we delay the onset of the mask relative to that of the target? One consequence of this delay will be that the observer has a longer time to view the target in isolation without its brightness being influenced by the darkness induction signal contributed by the mask. But when the mask does come on, the darkness induction signal that it generates will initially be particularly potent as a result of the transient component of the neural response to the mask edge and this will produce a transient darkening of the disk measured relative to the target brightness that would obtain if the target and mask were both left on indefinitely.

If we assume that the brightness percept is calculated at each infinitesimal moment in time, then we would expect the target to appear bright, then somewhat darker, then somewhat brighter. Whether the observer reports the target as being suppressed by the delayed mask or not would depend on when he or she reads out the target brightness from the neural code. We need to make an additional assumption about how the temporal readout occurs before we can make an unambiguous brightness prediction based on this dynamical brightness computation model. In what follows, we will assume that the target brightness is computed over a finite integration time that includes the period in which the target is viewed in isolation and at least some of the period in which the neural activations generated by the target and mask overlap in time (Bloch, 1885; Breitmeyer, 1984). This assumption seems reasonable because it would be optimal for the observer to report the target brightness without it being influenced by the brightness suppression introduced by the mask via the edge integration mechanism. But we know that the observer does not behave optimally: there is, in fact, some brightness suppression due to an interaction between the mask and the target.

Given this temporal linking hypothesis, we conclude that delaying the mask in time can only make the target more visible compared to the case where the SOA is zero, since the only effect of delaying the mask is to potentially reduce the percentage of the target integration time in which the neural response to the mask affects the target brightness. Thus, the U-shaped metacontrast masking function is not predicted from a model that combines transient and sustained neural activations with edge integration alone.

But, to this point, we have ignored the potential influence of contrast gain modulations acting between edges. It is these interactions that are proposed to be responsible for metacontrast masking. To predict the contribution of contrast gain control to the target brightness dynamics it seems reasonable to assume that it will also take some time for the contrast gain control originating from an edge to be felt at the location of the mask edge. The target onset is therefore expected to generate a spatially spreading contrast gain control signal having a “wave front” that travels outward from the target edge and modulates the gains of any active nearby edge detector neurons that it encounters. Through a secondary action, this spreading gain control signal will, according to the edge integration model, modulate the amplitudes of any lightness or darkness induction signals that are generated by these nearby edge detector neurons. Based on our previous experiments with static brightness matching displays (Popa & Rudd, in preparation; Rudd & Popa, 2007), we anticipate that the contrast gain control signal will act either to amplify or attenuate these induction signals, depending on the particular combination of contrast polarities of the interacting edges. This may sound like a vague prediction, but the direction of the gain modulation – either amplifying or attenuating – can be predicted on the basis the results of our past modeling of brightness matches performed with static displays composed on these same combinations of inner and outer edges (Popa & Rudd, in preparation; Rudd & Popa, 2007).

For the combination of target and mask edge contrast polarities considered here, the gain control acting from the target edge onto the mask edge is known from our past work to be amplifying and the gain control acting from the mask edge onto the target edge is known to be attenuating. The edge integration model asserts that the target brightness is computed from a spatial sum of induction signals derived from these two edges, so a gain control acting either from the mask edge to the target edge or from the target edge to the mask edge would be expected to influence the target brightness.

The transient activity generated by neural edge detector units at edge onset will be inherited by any gain control modulation that is exerted by those units onto other, nearby, edge detector neurons. Thus, the spreading gain control wave front should also exhibit a wave crest, which will produce either a transient increase or a transient decrease in the gain of any edge detector that it encounters. The transient gain modulation produced by this traveling wave crest will be in the same direction as the sustained gain change (i.e., either amplifying or attenuating), but of greater magnitude. Since the influence of contrast gain control takes time to spread between neural edge detector units, the effect of this transient boost in gain modulation strength will be to produce a time-delayed transiently-enhanced amplification or attenuation of any lightness or darkness induction signals that are generated by the nearby edge detector units. The time delay corresponding to the peak gain modulation will increase with increasing spatial separation between the gain-modulating edge and the gain-modulated edge.

The results of our previous experiments with static DAR displays lead us to expect that the gain control that operates from a light-inside target border to a dark-inside mask border will amplify, rather than attenuate, the strength of the darkness induction signal originating from the mask border (Popa & Rudd, in preparation; Rudd & Popa, 2007). We expect this to be the case because the strength of the darkness induction signal associated with an outer ring border increases when either the contrast of an inner ring border is increased or the borders are moved closer together by decreasing the width of the surround ring (Popa & Rudd, in preparation; Rudd & Popa, 2007). This behavior could account for the U-shaped masking metacontrast masking function in the following way. Suppose that the transient gain amplification of the darkness induction signal originating from the mask edge occurs at the same time that the neurons responding to the mask edge are exhibiting the regular transient activation that occurs at mask edge onset. These two transient amplification effects will combine multiplicatively (because gain control interactions are multiplicative by nature) to produce a particularly potent amplification of the darkness induction signal originating from the mask edge. This potent darkness induction signal will then sum with the lightness induction signal from the target edge to determine the target brightness, according to the basic assumption of the edge integration model. Note that the multiplicative “double-whammy” amplification of the darkness induction signal will only occur if the mask onset is delayed with respect to that of the target onset by the right time interval. Thus, when the mask edge is delayed relative to the target edge by the right interval, we expect that a brightness suppression of the target (i.e., metacontrast masking) will result.

In order for the double-whammy darkness induction signal amplification to explain metacontrast, it is necessary is that the gain increase applied to the mask edge by the double-whammy is more than sufficient to compensate for any tendency for the target brightness to be spared from temporally integrating with the darkness-inducing mask edge as a result of the target-mask delay. We assume that during part of the visual integration time the target is presented in isolation, and thus would normally appear bright, but during the rest of the visual integration time, the lightness and darkness induction signals elicited by target and mask edges overlap in time and bind spatially through the mechanism of edge integration to determine the overall target brightness. Given the appropriate temporal delay, the darkness that is induced in the target by the mask edge during the time that the target and mask bind spatially is sufficiently potent – as a result of the delayed contrast gain control effect – that the overall integrated brightness signal is smaller than it would be if the target and mask were presented either simultaneously (short SOA) or with a large temporal separation (long SOA). In the latter case, of course, the target and mask will not bind spatially at all.

**Figure 5.**
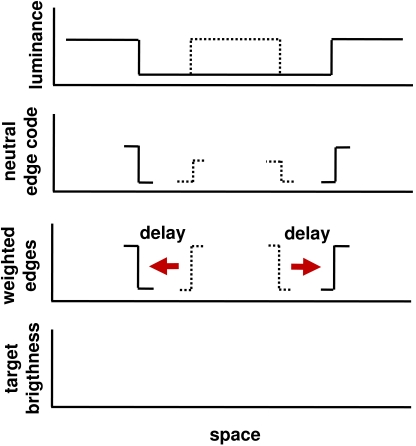
Proposed explanation of metacontrast based on edge integration and contrast gain control. The top graph in the figure shows the luminance profile of the stimulus. The target outline is indicated by a dotted line to signify that the target appears in an earlier frame than the mask (solid line). The presentation of the mask activates neural edge encoding units having the appropriate contrast polarity sensitivities and receptive fields at the locations of the mask edges (solid lines in the second graph). During the period in which the mask is presented, there may also be persisting activations in the edge encoding neurons that were activated by the target edges (dotted lines in the second graph). Both types of neural activations will potentially contribute to the target brightness, to a degree that depends on the edge weights. The third graph illustrates a case in which the weighted values of the neural activations corresponding to the target and mask edges happen to be identical. The edge weights are affected by two different processes. First, the target brightness computation algorithm tends to weight the target edge more heavily than it weights the more distant mask edge, all other things being equal. Second, a time-delayed contrast gain modulation acting from the target edge onto the mask edge will tend to boost the weight applied to the mask edge, with a particularly strong transient boost occurring at the optimal delay for metacontrast. In the hypothetical case illustrated, the darkness-inducing effect of the mask edge exactly cancels that lightness-inducing effect of the target edge, which results in the target brightness being neither higher nor lower than that of its immediate surround; thus, the target is made invisible. More generally, the target brightness may be modulated to a variable degree by the contrast gain control mechanism, with the largest target suppression effect occurring at the optimal SOA for metacontrast masking. If the contrast polarity of the mask edge is reversed, as in Weisstein's 1971 masking study, the transient gain modulation is attenuating, rather than amplifying (Rudd & Popa, in press). Since the presence of the mask edge in that case tends to lighten, rather than darken, the target, the transient attenuation of the lightness induction signal generated by the mask edge will also result in metacontrast masking.

In Figure 5 is presented a diagram illustrating how Type B metacontrast masking is produced by the dyna-mics of edge integration and contrast gain control in the case just discussed, in which an incremental target disk is followed in time by a larger dark masking disk (where a “dark” masking disk here means dark relative to the larger surround or background field). To my knowledge, this experiment has not been performed and thus the theory makes a novel prediction: that metacontrast masking should occur with this display. We next derive the model predictions for an experiment which has been performed; that is, the experiment of Weisstein (1971) mentioned earlier, in which a light target disk is masked by the delayed onset of a larger light masking disk. 

In Weisstein’s experiment, the target and mask borders both had contrast polarities of the light-inside type. For this combination of edge contrast polarities, the target and mask edges should both make a positive contribution to the target brightness as a result of edge integration. At short SOAs, the target should appear particularly bright as a result of the transient activation of neurons that encode the contrasts of the target and mask edges. This transient activation is expected to dissipate over time, resulting in a monotonic decrease in the target brightness, if the potential contribution of gain control interactions occurring between the target and mask edges is neglected.

Next consider the effects of adding the contrast gain control. For static disk-and-ring stimuli in which both edges are light-inside, we have shown in our previous work (Popa & Rudd, in preparation; Rudd & Popa, 2007) that the contrast gain control acting from the disk edge onto the outer ring edge in a DAR display acts to attenuate the lightness induction signal generated by the outer edge. In the Weisstein paradigm we would thus expect to see, given an appropriate time delay between the target and the mask, a transient *suppression* of the lightness inducing effect of the mask edge on the target brightness. Again, the gain control dynamics, when combined with the basic assumption of edge integration, predict the U-shaped masking function that is the hallmark of metacontrast masking. The gain control model thus predicts that metacontrast should be observed regardless of the contrast polarity of the mask edge.

The theory also predicts that there should be forward brightness modulation effects analogous to the backward masking effects already described. Such effects have been previously studied and are known as paracontrast masking (Breitmeyer, 1984; Breitmeyer & Öğmen, 2006; Breitmeyer, Kafaligonul, Öğmen, Mardon, Todd, Siegler, 2006). According to the model, paracontrast masking results from gain control processes that are initiated by the onset of the mask and act, after a time delay, to modulate the gain applied to the target edge. On the basis of our parametric model fits to DAR brightness matching data from experiments with static displays (Popa & Rudd, in preparation; Rudd & Popa, 2007), we expect these forward masking effects to be brightness enhancing, rather than brightness suppressing, when the target and mask each comprise a single edge. This prediction holds regardless of the contrast polarity mask edge, as long at the target disk is a luminance increment with respect to its immediate surround. A full justification for this claim is given in an upcoming paper (Popa & Rudd, in preparation). It is not yet clear how the magnitudes of the transient forward and backward brightness modulation effects might be expected to compare, but it seems likely that definite predictions regarding the relative magnitudes of the forward and backward brightness effects could be made on the basis of the parameter estimates obtained from fitting the model to brightness matches made with static stimuli.

In a recent study, Breitmeyer et al. (2006) studied paracontrast masking using a stimulus consisting of a dark disk target surrounded by a dark masking ring, with a spatial gap between the disk and ring. Their experimental results suggest that paracontrast consists of at least three separate effects: one involving excitation and two involving inhibition. As stated above, a stimulus containing a disk and a surround ring that is separated from the target disk by a spatial gap includes three edges and thus is expected to elicit considerably more complex gain control interactions than would the single-target-edge, single-mask-edge stimuli discussed above. It would not be surprising to discover that the former stimulus could generate three or more gain modulation effects having different time courses. But specific predictions remain to be worked out.

## References

[R1] Agostini T., Bruno N. (1996). Lightness contrast in CRT and paper-and-illuminant
						displays.. Perception & Psychophysics.

[R2] Aiba T. S., Stevens S. S. (1964). Relation of brightness to duration under light- and
						dark-adaptation.. Vision Research.

[R3] Alpern M. (1952). Metacontrast: historical introduction.. American Journal of Optometry.

[R4] Alpern M. (1953). Metacontrast.. Journal of the Optical Society of America.

[R5] Alpern M. (1963). Simultaneous brightness contrast for flashes of different
						durations.. Investigative Ophthalmology.

[R6] Arend L. E., Buehler J. N., Lockhead G. R. (1971). Difference information in brightness perception.. Perception & Psychophysics.

[R7] Arend L. E., Spehar B. (1993a). Lightness, brightness and brightness contrast: I. Illumination
						variation.. Perception & Psychophysics.

[R8] Arend L. E., Spehar B. (1993b). Lightness, brightness and brightness contrast: II. Reflectance
						variation.. Perception & Psychophysics.

[R9] Arrington K. F. (1994). The temporal dynamics of brightness filling-in.. Vision Research.

[R10] Bartels A., Zeki S. (2000). The architecture of the colour centre in the human visual brain:
						new results and a review.. European Journal of Neuroscience.

[R11] Becker M. W., Anstis S. (2004). Metacontrast masking is specific to luminance
						polarity.. Vision Research.

[R12] Bindman D., Chubb C. (2004a). Brightness assimilation in bullseye displays.. Vision Research.

[R13] Bindman D., Chubb C. (2004b). Mechanisms of contrast induction in heterogeneous
						display.. Vision Research.

[R14] Bloch A. M. (1885). Experience sur la vision. C. R. Seances Soc.
						Biol.. Fil..

[R15] Boden M. (2006). Mind as machine: A history of cognitive science..

[R16] Boynton R. M., Rosenblith W. A. (1961). Some temporal factors in vision.. Sensory communication.

[R17] Breitmeyer B. G. (1978a). Metacontrast as a function of mask energy.. Bulletin of the Psychonomic Society.

[R18] Breitmeyer B. G. (1978b). Metacontrast with black and white stimuli: evidence for
						inhibition of on and off sustained activity by either on or off transient
						activity.. Vision Research.

[R19] Breitmeyer B. (1984). Visual masking: An integrative approach..

[R20] Breitmeyer B. G., Ganz L. (1976). Implications of sustained and transient channels for theories of
						visual pattern masking, saccadic suppression and information
						processing.. Psychological Review.

[R21] Breitmeyer B. G., Kafaligonul H., Öğmen H., Mardon L., Todd S., Siegler R. (2006). Meta- and paracontrast reveal differences between contrast- and
						brightness-processing mechanisms.. Vision Research.

[R22] Breitmeyer B. G., Öğmen H. (2006). Visual masking: Time slices through conscious and unconscious
						vision..

[R23] Bressan P., Actis-Grosso R. (2001). Simultaneous lightness contrast with double
						increments.. Perception.

[R24] Broca A., Sulzer D. (1902). La sensation lumineuse en fonction du temps.. Journal de Physiologie et de Pathologie
						Générale.

[R25] Broca A., Sulzer D. (1904). La sensation lumineuse en fonction du temps.. Journal de Physiologie et de Pathologie
						Générale.

[R25a] Chevreul M.-E. (1839/1967). The principles of harmony and contrast of colours, and their
						applications to the arts..

[R26] Clarke S., Walsh V., Schoppig A., Assal G., Cowey A. (1998). Colour constancy impairments in patients with lesions of the
						prestriate cortex.. Experimental Brain Research.

[R27] Cohen M. A., Grossberg S. (1984). Neural dynamics of brightness perception: features, boundaries,
						diffusion, and resonance.. Perception & Psychophysics.

[R28] Cole R. E., Diamond A. L. (1971). Amount of surround and test inducing separation in simultaneous
						brightness contrast. Perception & Psychophysics.

[R29] Cornelissen F. W., Wade A. R., Vladusich T., Dougherty R. F., Wandell B. A. (2006). No function magnetic resonance imaging evidence for brightness
						and color filling-in in early human visual cortex.. Journal of Neuroscience.

[R30] Crawford B. H. (1947). Visual adaptation in relation to brief conditioning
						stimuli.. Proceedings of the Royal Society of London, Series B.

[R31] Diamond D. (1953). Foveal simultaneous contrast as a function of inducing- and
						test-field luminances.. Journal of Experimental Psychology.

[R32] Diamond D. (1955). Foveal simultaneous contrast as a function of inducing-field
						area.. Journal of Experimental Psychology.

[R33] Dunn B., Leibowitz H. (1961). The effect of separation between test and inducing fields on
						brightness constancy.. Journal of Experimental Psychology.

[R34] Fehrer E., Smith E. (1962). Effects of luminance ratio on masking.. Perceptual and Motor Skills.

[R35] Francis G. (2000). Quantitative theories of meta-contrast masking.. Psychological Review.

[R36] Francis G., Cho Y. S., Öğmen H., Breitmeyer B. G. (2006). Computational models of masking.. The first half second: The microgenesis and temporal dynamics of
						unconscious and conscious visual processes.

[R37] Francis G., Herzog M. (2004). Testing quantitative models of backward masking.. Psychonomic Bulletin & Review.

[R38] Friedman H. S., Zhou H., von der Heydt R. (2003). The coding of uniform colour figures in monkey visual
						cortex.. Journal of Physiology (London).

[R39] Fry G. (1934). Depression of the activity aroused by a flash of light by
						applying a second flash immediately afterwards to adjacent areas in the
						retina.. American Journal of Physiology.

[R40] Gerrits H. J. M., de Haan B., Vendrik A. J. H. (1966). Experiments with stabilized retinal images: relations between the
						observations and neural data.. Vision Research.

[R41] Gerrits H. J. M., Timmermann G. J. M. E. N. (1969). The filling-in process in patients with retinal
						scotoma.. Vision Research.

[R42] Gerrits H. J., Vendrik A. J. (1970). Simultaneous contrast, filling-in process and information
						processing in man’s visual system.. Experimental Brain Research.

[R43] Gilbert C.D., Wiesel T. N. (1989). Columnar specificity of intrinsic horizontal and corticocortical
						connections in cat visual-cortex.. Journal of Neuroscience.

[R44] Gilchrist A. L. (1988). Lightness contrast and failures of contrast: a common
						explanation.. Perception & Psychophysics.

[R45] Gilchrist A., Kossyfidis C., Bonato F., Agostini T., Cataliotti J., Li X. (1999). An anchoring theory of lightness perception.. Psychological Review.

[R45a] Goethe J. W. von, Eastlake C. L. (1810/1970). Theory of colours.

[R46] Grinvald A., Lieke L. L., Frostig R. D., Hildesheim R. (1994). Cortical point-spread function and long-range lateral
						interactions revealed by real-time optical imaging of macaque monkey primary
						visual cortex.. Journal of Neuroscience.

[R47] Grossberg S., Mingolla E. (1985). Neural dynamics of form perception: boundary completion, illusory
						figures, and neon color spreading.. Psychological Review.

[R48] Grossberg S., Todorovic D. (1988). Neural dynamics of 1-D and 2-D brightness perception: a unified
						model of classical and recent phenomena.. Perception & Psychophysics.

[R49] Hart W. M. Jr., Moses R. A., Hart W. M. (1987). The temporal responsiveness of vision.. Adler’s physiology of the eye: Clinical application..

[R50] Haynes J. D., Lotto R. B., Rees G. (2004). Responses of human visual cortex to uniform
						surfaces.. Proceedings of the National Academy of Sciences USA.

[R51] Heinemann E. G. (1955). Simultaneous brightness induction as a function of inducing- and
						test-field luminances.. Journal of Experimental Psychology.

[R52] Heinemann E. G., Jameson D., Hurvich L. (1972). Simultaneous brightness induction.. Handbook of sensory physiology.

[R52a] Hering E., Hurvich L. M., Jameson D. (1874/1964). Outlines of a theory of the light sense..

[R52b] Hess C., Pretori H., Flock J., Tenny J. H. (1884/1970). Quantitative investigation of the lawfulness of simultaneous
						brightness contrast.. Perceptual and Motor Skills.

[R53] Hirsch J. A., Gilbert C. D. (1991). Synaptic physiology of horizontal connections in the
						cat’s visual cortex.. Journal of Neuroscience.

[R54] Hong S. W., Shevell S. K. (2004). Brightness induction: unequal spatial integration with increments
						and decrements.. Visual Neuroscience.

[R55] Hubel D. H., Wiesel T. N. (1959). Receptive fields of single neurons in the cat’s
						striate cortex.. Journal of Physiology (London).

[R56] Hubel D. H., Wiesel T. N. (1968). Receptive fields and functional architecture of monkey striate
						cortex.. Journal of Physiology (London).

[R57] Hubel D. H., Wiesel T. N. (1977). Functional architecture of macaque visual cortex.. Proceedings of the Royal Society of London, Series B.

[R58] Hung C. P., Ramsden B. M., Chen L. M., Roe A. W. (2001). Building surfaces from borders in Areas 17 and 18 of the
						cat.. Vision Research.

[R59] Hurvich L. M. (1981). Color vision..

[R60] Jacobsen A., Gilchrist A. (1988). Hess and Pretori revisited: resolution of some old
						contradictions.. Perception & Psychophysics.

[R61] Jameson D., Hurvich L. M. (1964). Theory of brightness and color contrast in human
						vision.. Vision Research.

[R62] Kapadia M. K., Westheimer G., Gilbert C. D. (2000). Spatial distribution of contextual interactions in primary visual
						cortex and in visual perception.. Journal of Neuroscience.

[R63] Kennard C., Lawden M., Morland A. B., Ruddock K. H. (1995). Color discrimination and color constancy are impaired in a
						patient with incomplete achromatopsia associated with prestriate
						cortical-lesions.. Proceedings of the Royal Society of London Series B.

[R64] Kentridge R. W., Heywood C. A., Cowey A. (2004). Chromatic edges, surfaces and constancies in cerebral
						achromatopsia.. Neuropsychologia.

[R65] Kinoshita M., Komatsu H. (2001). Neural representation of the luminance and brightness of a
						uniform surface in the macaque primary visual cortex.. Journal of Neurophysiology.

[R66] Kolers P. (1962). Intensity and contour effects in visual masking.. Vision Research.

[R67] Kolers P., Rosner B. S. (1960). On visual masking (metacontrast): dichoptic
						observations.. American Journal of Psychology.

[R68] Kozaki A. (1963). A further study in the relationship between brightness constancy
						and contrast.. Japanese Psychological Research.

[R69] Kozaki A. (1965). The effect of co-existent stimuli other than the test stimulus on
						brightness constancy.. Japanese Psychological Research.

[R70] Lachman R., Lachman J. L., Butterfield E. C. (1979). Cognitive psychology and information processing: An
						introduction..

[R71] Lamme V. A. (1995). The neurophysiology of figure-ground segregation in primary
						visual cortex.. Journal of Neuroscience.

[R72] Lamme V. A. F., Rodriguez-Rodriguez V., Spekreijse H. (1999). Separate processing dynamics for texture elements, boundaries and
						surfaces in primary visual cortex of the macaque monkey.. Cerebral Cortex.

[R73] Lamme V. A. F., Spekreijse H. (2000). Modulations of primary visual cortex activity representing
						attentive and conscious scene perception.. Frontiers in the Biosciences.

[R74] Lamme V. A., Super H., Spekreijse H. (1998). Feedforward, horizontal, and feedback processing in the visual
						cortex.. Current Opinion in Neurobiology.

[R75] Lamme V. A., Zipser K., Spekreijse H. (2002). Masking interrupts figure-ground signals in V1.. Journal of Cognitive Neuroscience.

[R76] Land E. H. (1977). The retinex theory of color vision.. Scientific American.

[R77] Land E. H. (1983). Recent advances in retinex theory and some implications for
						cortical computations: color vision and the natural image.. Proceedings of the National Academy of Science USA.

[R78] Land E. H. (1986). An alternative technique for the computation of the designator in
						the retinex theory of color vision.. Proceedings of the National Academy of Sciences USA.

[R79] Land E. H., McCann J. J. (1971). The retinex theory of vision.. Journal of the Optical Society of America.

[R80] Lee T. S., Mumford D., Romero R., Lamme V. A. F. (1998). The role of the primary visual cortex in higher level
						vision.. Vision Research.

[R81] Lefton L. A. (1973). Metacontrast: a review.. Perception & Psychophysics.

[R82] Leibowitz H., Mote F. A., Thurlow W. R. (1953). Simultaneous contrast as a function of separation between test
						and inducing fields.. Journal of Experimental Psychology.

[R83] Levine R., Didner R., Tobenkin N. (1967). Backward masking as a function of interstimulus
						distance.. Psychonomic Science.

[R84] MacEvoy S. P., Kim W., Paradiso M. A. (1998). Integration of surface information in primary visual
						cortex.. Nature Neuroscience.

[R85] May J. G., Grannis S. W., Porter R. J. Jr. (1980). The ‘lag effect’ in dichoptic
						viewing.. Brain and Language.

[R86] Mizobe K., Polat U., Pettet M. W., Kasamatsu T. (2001). Facilitation and suppression of single striate-cell activity by
						spatially discrete pattern stimuli presented beyond the receptive
						field.. Visual Neuroscience.

[R87] Paradiso M. A., Hahn S. (1996). Filling-in percepts produced by luminance
						modulation.. Vision Research.

[R88] Paradiso M. A., Nakayama K. (1991). Brightness perception and filling-in.. Vision Research.

[R89] Pessoa L., Thompson E., Noe A. (1998). Finding out about filling-in: a guide to perceptual completion
						for visual science and the philosophy of perception.. Behavioral and Brain Sciences.

[R89a] Popa D., Rudd M. E. A neural theory of edge integration and contrast gain control in
						achromatic color perception..

[R90] Qiu F. T., von der Heydt R. (2005). Figure and ground in visual cortex: V2 combines stereoscopic cues
						with Gestalt rues.. Neuron.

[R91] Raab D. (1962). Magnitude estimation of the brightness of brief foveal
						stimuli.. Science.

[R92] Reeves A. (1982). Metacontrast U-shaped functions derive from two monotonic
						processes.. Perception.

[R93] Reid R. C. Jr, Shapley R. (1988). Brightness induction by local contrast and the spatial dependence
						of assimilation.. Vision Research.

[R94] Rossi A. F., Paradiso M. A. (1996). Temporal limits of brightness induction and mechanisms of
						brightness perception.. Vision Research.

[R95] Rossi A. F., Paradiso M. A. (1999). Neural correlates of perceived brightness in the retina, lateral
						geniculate nucleus, and striate cortex.. Journal of Neuroscience.

[R96] Rossi A. F., Rittenhouse C. D., Paradiso M. A. (1996). The representation of brightness in primary visual
						cortex.. Science.

[R97] Rudd M. E. (2001). Lightness computation by a neural filling-in
						mechanism.. Proceedings of the Society of Photo-Optical Engineers.

[R98] Rudd M. E. (2003a). Progress on a computational model of human achromatic color
						processing.. Proceedings of the Society of Photo-Optical Instrumentation
						Engineers.

[R99] Rudd M. E. (2003b). Neural mechanisms of achromatic colour perception: filling-in,
						edge integration, and awareness [Abstract].. Perception (suppl.).

[R100] Rudd M. E., Arrington K. F. (2001). Darkness filling-in: a neural model of darkness
						induction.. Vision Research.

[R101] Rudd M. E., Popa D. (2004a). A theory of the neural processes underlying edge integration in
						human lightness perception [Abstract]. Journal of Vision.

[R102] Rudd M. E., Popa D. (2004b). Edge integration and edge interaction in achromatic color
						computation, [Abstract]. Journal of Vision.

[R103] Rudd M. E., Popa D. (2007). Stevens’ brightness law, contrast gain control, and
						edge integration in achromatic color perception: a unified
						model.. Journal of the Optical Society of America A: Image Science, and
						Vision.

[R104] Rudd M. E., Zemach I. K. (2004). Quantitative properties of achromatic color induction: an edge
						integration analysis.. Vision Research.

[R105] Rudd M. E., Zemach I. K. (2005). The highest luminance anchoring rule in achromatic color
						perception: some counterexamples and an alternative theory.. Journal of Vision.

[R106] Rudd M. E., Zemach I. K. (2007). Contrast polarity and edge integration in achromatic color
						perception.. Journal of the Optical Society of America A: Image Science, and
						Vision.

[R106a] Rudd M. E. Edge integration and anchoring in the perception of lightness,
						brightness, and brightness contrast..

[R107] Saito H., Fukada Y. (1986). Gain-control mechanisms in X-type and Y-type retinal ganglion-
						cells of the cat.. Vision Research.

[R108] Sasaki Y., Watanabe T. (2004). The primary visual cortex fills in color.. Proceedings of the National Academy of Sciences USA.

[R109] Shapley R., Reid R. C. (1985). Contrast and assimilation in the perception of
						brightness.. Proceedings of the National Academy of Sciences of the USA.

[R110] Schiller P. H., Smith M. C. (1968). A comparison of forward and backward masking.. Psychonomic Sciences.

[R111] Smithson H. E. (2005). Sensory, computational, and cognitive components of human colour
						constancy.. Philosophical Transactions of the Royal Society.

[R112] Spencer T. J., Shuntich R. (1970). Evidence for an interruption theory of backward
						masking.. Journal of Experimental Psychology.

[R113] Stainton W. H. (1928). The phenomenon of Broca and Sulzer in foveal
						vision.. Journal of the Optical Society of America.

[R114] Stettler D. D., Das A., Bennett J., Gilbert C. D. (2002). Lateral connectivity and contextual interactions in macaque
						primary visual cortex.. Neuron.

[R115] Stevens J. C., Hall J. W. (1966). Brightness and loudness as functions of stimulus
						duration.. Perception & Psychophysics.

[R116] Stevens J. C., Marks L. E., Killeen P., Uttal W. (1999). Stevens power law in vision: exponents, intercepts, and
						thresholds..

[R117] Stevens S. S. (1953). On the brightness of lights and loudness of
						sounds.. Science.

[R118] Stevens S. S. (1961). To honor Fechner and repeal his law.. Science.

[R119] Stevens S. S. (1966). Duration, luminance, and the brightness exponent.. Perception & Psychophysics.

[R120] Stevens S. S. (1967). Intensity functions in sensory systems.. International Journal of Neurology.

[R121] Stevens S. S. (1975). Psychophysics: Introduction to its perceptual, neural, and social
						prospects..

[R122] Stewart A. L., Purcell D. G. (1974). Visual backward masking by a flash of light: a study of U-shaped
						detection functions.. Journal of Experimental Psychology.

[R123] Stigler R., Aberhalden E. (1926). Die untersuchung des zeitlichten verluafes der optischen erregung
						mittels des metakontrastes.. Handbuch der biologischen Arbeitsmethoden.

[R124] Turvey M. T. (1973). On peripheral and central processes in vision: interences from an
						information-processing analysis of masking with patterned
						stimuli.. Psychological Review.

[R125] Vladusich T., Lucassen M. P., Cornelissen F. W. (2006). Edge integration and the perception of lightness and
						darkness.. Journal of Vision.

[R126] von der Heydt R., Friedman H. S., Zhou H., Pessoa L., De Weerd P. (2003). Searching for the neural mechanisms of color
						filling-in.. Filling-in: From perceptual completion to skill learning..

[R127] von der Heydt R., Zhou H., Friedman H. S., Behrmann M., Kimchi R., Olson C. R. (2003). Neural coding of border ownership: implications for the theory of
						figure-ground perception.. Perceptual organization in vision: Behavioral and neural
						perspectives..

[R128] Wallach H. (1948). Brightness constancy and the nature of achromatic
						colors.. Journal of Experimental Psychology.

[R129] Wallach H. (1963). The perception of neutral colors.. Scientific American.

[R130] Wallach H. (1976). On perception.

[R131] Walsh V. (1999). How does the cortex construct color?. Proceedings of the National Academy of Sciences USA.

[R132] Weisstein N. (1971). W-shaped and U-shaped functions obtained for monoptic and
						dichoptic disk-disk masking.. Perception & Psychophysics.

[R133] Weisstein N., Jameson D., Hurvich L. (1972). Metacontrast.. Handbook of sensory physiology.

[R134] Weisstein N., Growney R. (1969). Apparent movement and metacontrast: a note on
						Kahneman’s formulation.. Perception & Psychophysics.

[R135] Werner H. (1935). Studies on contour: I. Qualitative analyses.. American Journal of Psychology.

[R136] Werner H. (1940). Studies on contour strobostereoscopic phenomena.. American Journal of Psychology.

[R137] Whittle P., Gilchrist A. L. (1994). Contrast brightness and ordinary seeing.. Lightness, brightness, and transparency..

[R138] Whittle P., Challands P. D. C. (1969). The effect of background luminance on the brightness of
						flashes.. Vision Research.

[R139] Zeki S., Aglioti S., McKeefry D., Berlucchi G. (1999). The neurological basis of conscious color perception in a blind
						patient.. Proceedings of the National Academy of Sciences USA.

[R140] Zeki S., Marini L. (1998). Three cortical stages of colour processing in the human
						brain.. Brain.

[R141] Zemach I. K., Rudd M. E. (2007). Effects of surround articulation on lightness depend onthe
						spatial arrangement of the articulated region.. Journal of the Optical Society of America A: Image Science, and
						Vision.

[R142] Zhou H., Friedman H. S., von der Heydt R. (2000). Coding of border ownership in monkey visual
						cortex.. Journal of Neuroscience.

[R143] Zipser K., Lamme V. A., Schiller P. H. (1996). Contextual modulation in primary visual cortex.. Journal of Neuroscience.

